# The Contribution of Gut Microbiota–Brain Axis in the Development of Brain Disorders

**DOI:** 10.3389/fnins.2021.616883

**Published:** 2021-03-23

**Authors:** Jessica Maiuolo, Micaela Gliozzi, Vincenzo Musolino, Cristina Carresi, Federica Scarano, Saverio Nucera, Miriam Scicchitano, Francesca Oppedisano, Francesca Bosco, Stefano Ruga, Maria Caterina Zito, Roberta Macri, Ernesto Palma, Carolina Muscoli, Vincenzo Mollace

**Affiliations:** ^1^IRC-FSH Department of Health Sciences, University “Magna Græcia” of Catanzaro, Catanzaro, Italy; ^2^IRCCS San Raffaele, Rome, Italy

**Keywords:** gut microbiota, neurological disorders, gut microbiota-brain axis, enteric nervous system, anxiety and depression, autistic spectrum disorders, multiple sclerosis

## Abstract

Different bacterial families colonize most mucosal tissues in the human organism such as the skin, mouth, vagina, respiratory, and gastrointestinal districts. In particular, the mammalian intestine hosts a microbial community of between 1,000 and 1,500 bacterial species, collectively called “microbiota.” Co-metabolism between the microbiota and the host system is generated and the symbiotic relationship is mutually beneficial. The balance that is achieved between the microbiota and the host organism is fundamental to the organization of the immune system. Scientific studies have highlighted a direct correlation between the intestinal microbiota and the brain, establishing the existence of the gut microbiota–brain axis. Based on this theory, the microbiota acts on the development, physiology, and cognitive functions of the brain, although the mechanisms involved have not yet been fully interpreted. Similarly, a close relationship between alteration of the intestinal microbiota and the onset of several neurological pathologies has been highlighted. This review aims to point out current knowledge as can be found in literature regarding the connection between intestinal dysbiosis and the onset of particular neurological pathologies such as anxiety and depression, autism spectrum disorder, and multiple sclerosis. These disorders have always been considered to be a consequence of neuronal alteration, but in this review, we hypothesize that these alterations may be non-neuronal in origin, and consider the idea that the composition of the microbiota could be directly involved. In this direction, the following two key points will be highlighted: (1) the direct cross-talk that comes about between neurons and gut microbiota, and (2) the degree of impact of the microbiota on the brain. Could we consider the microbiota a valuable target for reducing or modulating the incidence of certain neurological diseases?

## Introduction

The mammalian intestine hosts a microbial community of approximately 1,000–1,500 bacterial species called the “microbiota,” destined to evolve over the course of the host’s life and over the generations and subject to environmental changes. It has been amply demonstrated that the composition of the intestinal microbiota is also influenced by diet, age, lifestyle, and the presence of inflammatory processes (Na et al., 2017; Kim and Jazwinski, 2018), so it is accurate to say that the composition of the microbiota differs substantially from individual to individual. The microbial genome sequences contain approximately 3 × 10^6^ genes, 150 times the length of the human genome. In addition, the commensal microorganisms that reside in the intestine exceed human somatic cells at a ratio of about 10:1 (Na et al., 2017). In healthy adults, the microbiota is primarily composed of five bacterial phyla: Firmicutes (79.4%), Bacteroidetes (16.9%), Actinobacteria (2.5%), Proteobacteria (1%), and Verrucomicrobia (0.1%) (Davenport et al., 2014). Normally, the gut microbiota consists of a high diversity and abundance of microbial populations, and this condition is known as “eubiosis.” Over the span of a lifetime, a wide range of factors, including an incorrect diet, sleep disorders, obvious pathological conditions, drug abuse, pharmacological therapy, and many others, can alter diversity and abundance of the microbiota leading to a state of “dysbiosis” (Iebba et al., 2016). The symbiotic relationship between the gut microbiota and the host organism has been described as mutually beneficial: the host provides the nutrients and a suitable habitat for the microbiota, while the gut microbiota supports the host’s intestinal development and maturation by providing nutrients. Therefore, a state of co-metabolism is generated between the microbiota and the host system (Obrenovich, 2018). Over the past 15 years, it has been highlighted how the microbiota is able to control and influence certain segments of the physiology of the host such as the immune system, the digestive system, and the brain (Morrison and Preston, 2016; Rooks and Garrett, 2016; Dinan and Cryan, 2017). For example, the microbiota plays a vital role in the formation of the host’s immune system, and it can be said that there is real cross-talk between these two districts, which allows the development of the host’s tolerance to the harmless antigens of the microbiota. Studies in germ-free animals (GF) have shown that the lack of the gut microbiota leads to significant deficiency in the functioning of the immune system (Belkaid and Harrison, 2017).

Until a few years ago, it was a common opinion that a fetus developed in a completely sterile uterine environment and that the first intestinal colonization occurred from birth onwards. However, recent studies have disproved this conception and have demonstrated the presence of microorganisms in the placenta, amniotic fluid, and umbilical cord (Aagard et al., 2014; Pelzer et al., 2017). It has been hypothesized that the fetus begins to colonize its own developing gastrointestinal tract by swallowing the amniotic fluid and the bacteria it contains in the uterus. In addition, fetal and newborn meconium contains microorganisms (Walker et al., 2017). It is only with childbirth, however, that infants are exposed to most of the microorganisms responsible for intestinal colonization and the development of the microbiota. Moreover, the type of delivery a newborn undergoes is very important since the initial intestinal microbiota of the baby could resemble, in terms of composition, the microorganisms with which it came into contact during delivery. For example, following a vaginal birth, the baby comes into contact with the vaginal microbiota, while following a caesarean section the child comes into contact with the epidermal microbiota (Shao et al., 2019). It has also been shown that babies born through natural childbirth could develop a more varied microbiota than babies born by cesarean section (Nagpal and Yamashiro, 2018; Jagodzinski et al., 2019). Despite microbial exposure *in utero*, most of the microorganisms that will colonize the infant’s intestine are acquired after childbirth. The initial colonization pattern is believed to be chaotic, and numerous studies suggest that environmental factors and diet are responsible for major changes in composition (Savage et al., 2018). In a child, during the first phase of intestinal colonization, the microorganisms present are predominantly aerobic, such as Enterobacteria, *Staphylococci*, and *Streptococci*, many of which have a pathogenic potential. Subsequently, microorganisms become predominantly anaerobic. The composition of the intestinal community continues to change during the first year of life and thereafter in response to external factors such as diet and the use of antibiotics (Hill et al., 2017). It has been highlighted that a significant difference in the composition of the baby’s intestinal microbiota occurs in relation to the type of milk he drinks, the type of weaning he undergoes, and the different types of foods he consumes (Brahm and Valdés, 2017). Breastfeeding (BF) is the nourishment conceived by nature for newborns and infants, although in the last decades, it is very frequently replaced with various milk formulations (formula-fed, FF). In general, it is possible to say that BF has proven to be a protective factor for many inflammatory bowel diseases as well as for neurodevelopment, while the use of the various types of milk formulated for children has been shown to increase the risk of intestinal diseases, following an incorrect formation of the intestinal microbiota (Le Doare et al., 2018). BF infants have a more uniform intestinal microbial population than FF infants. This aspect has very important implications for the future of the child: in fact, the study of the intestinal microbiota of a BF newborn could furnish fundamental information on the correct development of the immunitary system, the immune response and tolerance and for the tendency to develop fewer allergic, inflammatory, and autoimmune pathologies (Vieira Borba et al., 2018). The composition of breast milk includes proteins, fats and carbohydrates, as well as immunoglobulins, endocannabinoids, and indigestible polysaccharides. Some of these polysaccharides act as real prebiotics capable of selectively stimulating the growth of beneficial bacteria (Sayres and Visentin, 2018); most of these are Bifidobacteria, indispensable for strengthening the protection of the intestinal mucosa through their specific activity against pathogens and through the increase in immunoglobulin A, related to the modulation of the intestinal immune system. After weaning, the composition of the intestinal microbiota still varies in relation to the type of feeding, while after 3 years of life, in the absence of disturbances such as long-term dietary changes or the repeated use of antibiotics and drugs, the bacterial composition of the intestinal microbiota remains approximately stable until old age. In general, over the course of life, the Bifidobacteria decrease while the Bacteroidetes and Firmicutes increase (Derrien et al., 2019).

Much scientific evidence has suggested that the intestinal microbiota maintains bidirectional interaction with critical parts of the central nervous system (CNS) as well as the immunitary system through both direct and indirect pathways (Petra et al., 2015; Fung et al., 2017). In addition, intestinal microbiota dysbiosis has been closely linked to various diseases, such as obesity, type 2 diabetes mellitus, hypertension, necrotizing enterocolitis, and many inflammatory bowel diseases (Weiss and Hennet, 2017; Allaire et al., 2018). Moreover, the existence of a close correlation between the intestinal microbiota and the brain has become increasingly evident even though the mechanisms involved are not completely clear: the existence of a gut–brain axis has become the main focus of neuroscience (Cryan et al., 2019). Evidence that dysfunction of the microbiota can play a key role in the development of certain neurological diseases is provided by the discovery that intervention that restores microbiota health and integrity may have a positive influence on the course, symptoms, and clinical conditions of said diseases (Julio-Pieper et al., 2014; Patel and DuPont, 2015; Fiorentino et al., 2016; Boyton and Altmann, 2019; Garg et al., 2019). This is the main reason why the intestine is called the “second brain” (Ali et al., 2019). In this direction, it would be interesting to consider neurological disorders and pathologies related to neurodegeneration, not as of being of “neural origin,” but rather as being linked to other external factors, and the health of the intestinal microbiota could be one of these factors. In the light of what has been stated, it is clear how important the first phase of intestinal colonization is. A consecutive question is, “To what degree can proper intestinal colonization affect the possibility of developing neurological disorders?” Therefore, the main purpose of this review is to consider the alteration of the microbiota as a likely cause of numerous neurological and degenerative disorders.

In the following sections we will first deepen current knowledge on the dysfunction of the microbiota in several cerebral diseases, and their “non-neuronal origin”; later, we will compare these scientific data with the classical knowledge that identifies the brain as the primary cause of some specific disorders. Our attempt, as already mentioned, will be to shift the direction of the interpretation of these pathologies “from the microbiota to the brain” instead of “from the brain to the microbiota.”

## Neurological Disorders and Microbiota: From the Microbiota to the Brain

Neurological disorders are diseases of the central and peripheral nervous system that can impair the functioning of brain, spinal cord, cranial and peripheral nerves, autonomous nervous system, nerve roots, and neuromuscular plaque. The causes can be many: (a) diseases due to gene alteration; (b) degenerative diseases characterized by the progressive loss of populations of neurons that are selectively vulnerable; (c) diseases of blood vessels that may cause bleeding in the brain; (d) diseases due to problems in the development of the nervous system; (e) disorders due to injury to the spinal cord or brain; (f) convulsive disorders; (g) brain tumors; (h) more or less severe infections (Dugger and Dickson, 2017; Chi et al., 2018). Up to now, neurological disorders have always been considered to be a specific consequence of morphological and/or functional alterations of some neuronal segment. In this review, we will NOT consider them as such, but rather as the result of the alteration of the gut microbiota. Three neurological disorders will be explored below: anxiety–depression, autistic spectrum disorder (ASD), and multiple sclerosis. Despite the fact that these pathological conditions have completely different characteristics, they seem to have some points in common:

• involve the CNS;
• can present themselves in pathological form at a very early stage in life;
• are closely related to intestinal dysbiosis.

Can we identify intestinal dysbiosis as the actual cause of some of these neurological disorders? Could intestinal dysbiosis be considered to be the common denominator of the three neurological disorders that we are considering? We will try this approach with the help of current literary knowledge.

### Anxiety and Depression

Anxiety and depression are psychiatric and neurological disorders that occur in 25% of the global population. In addition, these two pathological states seem to be closely related: in fact, 90% of patients with anxiety disorders also develop depression and 85% of patients with depression show significant anxiety (Bui and Fava, 2017). The phenomena of anxiety and depression can occur as early as childhood or adolescence, as well as at any other time in an individual’s life. These two pathologies, both in early and late forms, considerably differ in terms of clinical symptoms (Groeneweg-Koolhoven et al., 2017). In recent decades, the increase in depressive symptoms has also led to an increase in the number of teenage deaths from suicide (Jorm et al., 2017; Matsumoto et al., 2017; Weinberger et al., 2018; Twenge et al., 2019). The states of anxiety and depression are constantly associated with changes in the composition and stability of the intestinal microbiota and this close correlation has been studied (Dinan and Cryan, 2013; Tognini, 2017; Zhao et al., 2018; Thomaz et al., 2019). In an important scientific study on animal models, it was found that the transfer of the microbiota from models with depression to that of other animals deprived of their microbiota also determined the transfer of the behavioral and physiological characteristics of the depression (Kelly et al., 2016). Since it is currently widely accepted that our intestinal microbiota is essential for brain processes (myelination, neurogenesis, microglia activation, and psychological processes such as mood and cognition) (Dinan and Cryan, 2016), the early formation of a well-balanced microbiota and its maintenance throughout life seems to reduce the occurrence of a wide variety of diseases, including behavioral and neuropsychiatric disorders (Cenit et al., 2017). Childhood and adolescence are the most dynamic and vulnerable periods in the life of an individual for developing and achieving the composition of the microbiota and certain events and conditions may be important contributors including diet, drugs, stress, and infections (Borre et al., 2014; Erny et al., 2015). Still, although the composition of the gut microbiota may vary in adulthood as a result of the effects of harmful or negative factors, the symbiotic link between the host and microbiota are established early in life (Desbonnet et al., 2015). Stress-related disorders encourage the increase of some bacterial species (Kelly et al., 2015). A study conducted on a group of healthy students showed that, following an extremely stressful period, fewer species of *Lactobacillus* were present in the stool. Moreover, a condition of chronic stress induced in mice reduced *Lactobacillus, Eubacterium rectale*, *Lachnospira, Butyricicoccus, Sutterella*, and *Faecalibacteria* and increased the number of pathogenic species *Clostridium*, Proteobacteria, Actinobacteria, and *Enterobacteriaceae* (Tian et al., 2019). An important and well-organized experimental work revealed that an altered composition of the intestinal microbiota induced in mice through the use of massive doses of antibiotics in a period corresponding to the early adolescence of the animals, led to alterations in cognitive function and the appearance of symptoms related to anxiety and depression (Zeraati et al., 2019). It has been suggested that the type of diet can have positive or negative effects on depression: in particular, human studies have shown that an inversely proportional correlation exists between the consumption of omega-3 polyunsaturated fatty acids and anxiety–depression; that is, the more the assumption of omega-3, the less anxiety–depression (Grosso et al., 2014; Oppedisano et al., 2020). Finally, treatment with different probiotics has been particularly effective in reducing depressive behavior in animal models. For example, the administration of a probiotic cocktail, composed of *Lactobacillus rhamnosus* and *Lactobacillus helveticus*, reduced their symptoms of depression and anxiety.

### Autism Spectrum Disorder

ASDs include a set of alterations in neurological development characterized by deficits in social interaction and communication, and repetitive and stereotyped behavior. ASD can vary in terms of symptomatic severity, varying from mild to very severe. The main symptoms of ASD appear as early as the first year of life and include delays in language development, repetitive movements, very few interests, limited or absent eye contact, limited sharing of feelings or interests, significant discomfort arising from a change in routine, failure to start and maintain conversations, strong attachments to specific objects, excessive reaction to sounds or visual cues, loss of interest in social relationships, and difficulty in engaging in imaginary play. To date, there is no specific treatment for these disorders and early medical behavioral therapy has been shown to improve but not resolve problems relating to mental capacity, language, and social ability (Howlin and Magiati, 2017; Lord et al., 2018). ASD is a pathology with an unclear and multifactorial etiology, yet several causes have been identified, which include genetic anomalies, dysregulation of the immune system, inflammatory processes, and interaction with environmental factors (Famitafreshi and Karimian, 2018).

ASD disorders are often associated with gastrointestinal co-morbidities and a large proportion of patients (23–70%) also suffer from constipation, diarrhea, abdominal pain, flatulence, and intestinal gas (Mulle et al., 2013). They are also associated with food restriction and eating problems (such as “selective and picky eaters” who show aversion to specific colors, textures, odors, or other food characteristics) (Cermak et al., 2010). The consequences of this are reduced dietary quality, nutritional deficiency, and altered composition of the intestinal microbiota (Berding and Donovan, 2016). Generally, the composition of the intestinal microbiota of autistic children shows substantial differences: the data in the scientific literature indicate overall a reduction of *Bacteroides* with a ratio (% ASD children/% control children) equal to 0.71; a reduction of *Bifidobacterium* with a ratio equal to 0.52; a reduction of *Escherichia coli* with a ratio of 0.3; an increase in *Faecalibacterium* with a ratio of 1.32; and an increase in *Lactobacillus* with a ratio of 2.17. The presence of *Clostridium* remains substantially unchanged (Tomova et al., 2015). Although it cannot be said that there are specific bacteria compatible and associated with the onset of ASD, it is clear that these neurological disorders are accompanied by lower levels of beneficial bacteria and higher levels of harmful bacteria (Iglesias-Vázquez et al., 2020). It has been hypothesized that the increase in *Faecalibacterium* in ASD children is responsible for the progression of inflammatory processes, with increased levels of type I interferon, and the alteration of the intestinal barrier. In addition, the reduction of *Bifidobacteria*, the main producers of lactic acid capable of suppressing the growth of pathogenic bacteria, leads to an alteration of the immunitary system (Hashemi et al., 2017). The reduction of *Bifidobacterium* also results in reduced levels of short-chain fatty acids (SCFAs), common in ASD children.

In ASD patients, an important correlation exists between the aforementioned active neuropeptides and disability. Their incorrect interaction involves a series of inflammatory disorders, autoimmune conditions, neurodegenerative and metabolic disorders, as well as problems regarding mood, behavior, cognitive function, autistic spectrum dysfunction, stress, and pain (Lerner et al., 2017). A likely mechanism could be that proteic transducers escape from the gastrointestinal tract and enter the bloodstream exerting a systemic effect (Mead and Ashwood, 2015).

The bacteria that make up the gut microbiota and their metabolites could play a critical role in the pathophysiology of ASD (Xu et al., 2019). In fact, some experiments have shown that patients who have had their intestinal microbiota remodeled through the administration of antibiotics or bacterial transfer therapy in the intestine, presented with attenuated symptoms of ASD (Kang et al., 2017). The administration of probiotics was sufficient to vary the composition of the microbiota and to guarantee greater control of the intestinal barrier (Doenyas, 2018). The mechanisms studied so far that correlate the intestinal microbiota with ASD disorders are manifold and concern the breakdown of the integrity of the intestinal barrier, the production of toxins, and the formation of intestinal dysbiosis (Ding et al., 2017). Another extremely important aspect concerns the increase in neurotoxins produced by the intestinal system of ASD children that act distally on the brain (Yang and Chiu, 2017). It is also important to highlight the fact that the microbiota and the metabolites formed by it are indispensable for the maintenance of cerebral white matter and the integrity of the blood–brain barrier (Golubeva et al., 2017).

### Autoimmunity and Multiple Sclerosis

Innate immunity is the host’s first defensive line for eliminating invading and foreign pathogens. Through this type of immunity, in fact, critical mechanisms are activated for the rapid detection and elimination of pathogens. This type of immunity does not have immune memory and can only be based on specific receptors, which have been selected during the evolutionary process and which can only bind to the same unchanged antigens. Conversely, adaptive immunity has evolved with the aim of providing a vast repertoire of antigenic recognition of self- and non-self-molecules. Adaptive immunity uses the strictly regulated interaction between the cells presenting the antigen and the B and T lymphocytes. These cells consequently activate the immunological effector pathways in order to contrast the specific pathogen. In addition, adaptive immunity has an immunological memory capable of recognizing an antigen that has already been encountered and destroying it (Vatner and Janssen, 2019). Autoimmunity occurs when the immune system loses self-tolerance and begins to counteract its own molecules and cells. If this characteristic of immunological imbalance persists constantly in the body, more or less serious autoimmune diseases develop (Stetson, 2018). Multiple sclerosis (MS) is a demyelinating autoimmune disease of the nervous system and is characterized by chronic inflammation, breakdown of the BBB, and infiltration of immune cells into the CNS. The latter lead to the destruction of the myelin sheath with axonal loss and progressive disability (Matveeva et al., 2018; Maiuolo et al., 2019a,b). In general, it has been shown, in fact, that in MS, the inflammatory process involves T lymphocytes, CD4 and CD8, B lymphocytes, activated monocytes, and astrocytes. Oxidative stress was also a key factor in the pathogenesis of MS: in particular, macrophages and microglia produce reactive oxygen species (ROS) and reactive nitrogen species (RNS) and secrete pro-inflammatory cytokines. These conditions develop neurodegeneration and excitotoxicity (Corasaniti et al., 2007; Oppedisano et al., 2020). It is clear, therefore, that MS is a multi-factorial pathology and genetic, environmental, and immunological factors are included in its etiology. Multiple sclerosis appears particularly in young women (female:male ratio = 3:1), especially in those women who have suffered intestinal disorders since birth (Sauma and Casaccia, 2020). It has been recently shown that an alteration of the intestinal microbiota leads to over-stimulation of immune cells with a higher incidence of the development of autoimmune diseases such as rheumatoid arthritis, systemic lupus erythematosus, and MS (Brown et al., 2019). In particular, there is increasing evidence (found in animal models) according to which there is a relationship between the type of intestinal microflora and the progression of MS. According to these scientific data, autoimmune reactions can be produced by molecular mimicry and by the excessive production of lymphocytes (Chu et al., 2018). Compared to healthy controls, patients with MS show a decrease in the proportion *of Faecalibacterium* and *Fusobacterium* and an increase in *Escherichia, Shigella, Clostridium, Eubacterium rectal, Corynebacterium*, and Firmicutes (Tremlett et al., 2016a,b,c; Tremlett and Waubant, 2017). Some metabolic by-products of the intestinal microflora activate the transcription of the gene *foxp3*, responsible for the codification of the FOXP3 protein, a transcriptional regulator also known as scurfin; FOXP3 binds to the promoters of the genes involved in the development and regulation of T-cell receptors, promoting the attenuation of the immune response (Zhang et al., 2019). These microbiota by-products include SCFAs, responsible for activating the FOXP3 pathway and modulating the immune response. When intestinal dysbiosis occurs, this whole regulatory process decays and pathways that lead to autoimmunity are triggered (Khan and Ghazanfar, 2018). In some scientific papers, it has been shown that Bacteroidetes, present in the intestinal microbiota, produce lipid 654, which behaves as a ligand for human and mouse Toll-like receptor 2 (TLR2), a toll-like receptor with a role in the immunitary system (Selmi, 2017). An important scientific study showed that lipid 654 was present in the serum of all the healthy subjects examined. Conversely, extremely low lipid levels were found in MS patients, indicating, for the first time, this lipid as a serum biomarker of MS (Farrokhi et al., 2013; Browne et al., 2019). There are some bacteria, such as *Clostridium perfringens*, which produce natural toxins that are involved in the early stages of MS (Wagley et al., 2019). These toxins are absorbed by the intestine, enter the bloodstream, and cause symptoms typical of those that occur in more or less the early stages of MS, such as blurred vision, lack of coordination, or spastic paralysis (Tsunoda, 2017). The suspicion that these toxins could be the potential cause of MS was already described in the 90s, since man is not a natural host of *C. perfringens*, but becomes so in the case of intestinal dysbiosis, which allows this bacterial family to gain the upper hand (Savva et al., 2019). The migration of these toxins to the CNS occurs precisely as a result of their binding to the receptors present in the vascular system and, in this way, they are conveyed to the myelinated and non-myelinated areas of the brain (Anwar et al., 2019). Experimental MS patients showed an exacerbation of their intestinal balance following the administration of first- and second-line drugs recommended for this pathology. This worsens the picture of the already-compromised microbiota. For example, the administration of the drug Glatiramer Acetate induces a reduction in the “good” *Bacteroidaceae, Faecalibacterium, Lactobacillaceae*, and *Clostridium* as compared to untreated patients (Abdurasulova et al., 2018). BF, as already mentioned, provides the child with a fundamental matrix of immune information regarding the formation of his microbiota. The data available in literature show a clear link between BF and the reduced development of some autoimmune diseases such as MS, diabetes, and celiac disease (Vieira Borba et al., 2018). However, further scientific studies are needed to understand the mechanisms behind this link. To date, this correlation is also supported by the discovery that in patients with MS the administration of specific probiotics manages to increase several bacterial taxa of the intestinal microbiota that are normally reduced and/or absent. At the immune level, the administration of specific probiotics induces an anti-inflammatory response with the consequent reduction of inflammatory cells, such as monocytes and dendritic cells (Tankou et al., 2018).

## Classical Knowledge of Anxiety–Depression, ASD, and MS: Brain Origin of Diseases

Neurodegenerative diseases are characterized by progressive dysfunction and loss of neurons, which leads to the distinct involvement of a particular functional system. In normal physiological conditions, the death of neuronal cells is mainly limited to the result of aging. In fact, mature neurons have the ability to manage and overcome different stressful conditions in order to maintain cellular homeostasis (Kole et al., 2013; Maiuolo et al., 2020). However, in diseases, the loss of specific neurons of the brain is a fundamental pathological characteristic (Kovacs, 2017) and cell death is the final destiny for a neuron that has accumulated more stressful events than it can recover from: this condition is commonly present in neurodegenerative diseases (Hollville et al., 2019).

For this reason, neurodegenerative diseases can be classified according to (1) the clinical characteristics they present; (2) the anatomical distribution of the neurodegeneration in act; and (3) the main molecular abnormalities encountered (Chi et al., 2018). A common element of many neurodegenerative diseases are aberrant protein aggregates, and their location and composition vary in different diseases (Dugger and Dickson, 2017). Loss of neurons can be appreciated in most neurodegenerative diseases, including Alzheimer’s disease (AD), Parkinson’s disease (PD), Huntington’s disease, and amyotrophic lateral sclerosis (SLA). Until now, anxiety–depression, AD, and MS have all been considered to be disorders, the onset of which is mainly neurological in origin and have been addressed and treated as such. Below, we will describe our principal knowledge of these pathological disorders, the recommended therapies, and the scientific limitations known so far. Anxiety and depression are considered neuropsychiatric disorders and are found in normal cerebral conditions—that is, in the absence of morphological alterations—yet with reduced activity (Marwood et al., 2018). Although the causes of anxiety–depressive disorders are not yet known, it is believed that stress and genetic predisposition are essential factors. In general, stress activates the adrenal glands and leads to the overproduction of cortisol, which chronically stimulates certain brain structures such as the hippocampus and amygdala. This hyperstimulation reduces the volume and functionality of these districts by favoring the onset of the symptoms of anxiety and depression (Fiksdal et al., 2019). Some scientific studies have reported volumetric modifications of parts of the brain in patients suffering from anxiety–depressive symptoms: conducted measurements through nuclear magnetic resonance (NMR) and positron emission tomography (PET) showed a reduction of the amygdala–hippocampus complexes and prefrontal cortexes of these patients as compared to the control group (Gupta et al., 2019). Following pharmacological therapy, the morphological and functional recovery of the aforementioned anatomical structures was found, and in animal models, the antidepressant therapy determined the multiplication of stem cells in the hippocampus and in the amygdala (Ebrahimi-Ghiri et al., 2019). Nevertheless, it is important to emphasize that pharmacological therapy does not completely solve anxiety–depressive symptoms (Akil et al., 2018). The anxiety-depressive syndrome is considered prodromal for numerous neurological diseases of degenerative, inflammatory, or vascular nature: in fact, patients suffering from “neurological depression” may develop these diseases more frequently than the general population. Some epidemiological studies have shown the existence of a bi-directional relationship between neurological disorders and depressive disease: PD, AD, and epilepsy are often preceded by episodes of anxiety–depression (Jacobson and Newman, 2017; Pede et al., 2017; Steffens, 2017). Therefore anxiety–depression can be considered to be a risk factor for neurological disorders. To date, behavioral–cognitive therapy is the first-line treatment for depression and anxiety disorders, although it has been shown to be ineffective in 50% of patients (Cuijpers et al., 2014; Leuzinger-Bohleber et al., 2019), since few patients receive high-quality therapy. In fact, most affected people obtain non-optimal results in terms of inadequate dosages, the appearance of side effects, and interaction with drugs taken for other diseases. Many are not treated at all (David and Gourion, 2016). Psychological intervention through drug therapy is particularly recommended, and these associated therapies have shown benefits for the treatment of both depression and anxiety (Tolin, 2017; Marwood et al., 2018).

As has already been described, ASD is a pathology with an unclear and multifactorial etiology: in fact, among the causes considered to date, there are genetic abnormalities, dysregulation of the immune system, inflammatory processes, and interaction with environmental factors. Precisely for this reason, the diagnosis of this disorder is particularly problematic. Neural systems, involved in ASD, include the upper right temporal area, amygdala, prefrontal cortex, hippocampus, and Broca area, responsible for emotions, memory motor coordination, phonological processing, and executive functions. It follows that a certain vulnerability in the socio-behavioral system may be a risk factor for ASD (Ecker et al., 2015). To date, there is no specific treatment for these disorders and early medical behavioral therapy has been shown to improve, but not solve, deficits in mental capacity and linguistic and social abilities (Howlin and Magiati, 2017; Lord et al., 2018). In addition, support services are overburdened or insufficient (Shattuck et al., 2012; Hollocks et al., 2019). ASD not only involves affected patients and their families but also has an economic impact on overall spending: in fact, there are significant direct costs associated with ASD, which include expenses related to provision of special education, housing, and medical care programs, and indirect costs such as loss of productivity affecting individuals with ASD (Buescher et al., 2014). At present, the prevailing practice follows a pattern of “wait and see” (whether delays worsen the situation or allow it to resolve itself) or “wait to fail” (identification occurs when ASD is established). It has been shown that over 50% of children with ASD do not receive a correct diagnosis before the sixth year of age and today scientific knowledge is aimed at determining a more precocious intervention (Baio et al., 2018). For most people, the obvious symptoms of ASD will not become apparent until childhood or later with the first problems being reported at around 32 months of life. Differences in social communicative characteristics will gradually emerge during the second year, so it would be difficult to act more quickly than the known practice (Estes et al., 2015; Sacrey et al., 2019). The North American Prodrome Longitudinal Study (NAPLS) has considered the possibility that young patients with ASD may develop a full-blown psychosis during their lifetime. The results obtained showed a correlation between ASD and psychosis in 18% of patients with ASD. However, due to the difficulty of the study, which tends to follow enrolled patients for a long time, there is a need to expand research (Foss-Feig, 2019).

Multiple sclerosis, as has already been said, is an autoimmune inflammatory disease that affects the CNS, brain, and spinal cord, characterized by demyelination and axonal loss. Although the etiology of MS remains unknown it is a common opinion that the disease is caused by an immune dysregulation triggered by genetic and environmental factors. The loss of function of the axons classifies MS as both a progressive and degenerative disease. Current pharmacological therapy includes the administration of anti-inflammatory corticosteroids, immunomodulators, or humanized monoclonal antibodies, all of which could help to alter the course of the disease. In summary, we can say that significant progress has been made in the area of MS therapy, but testing should continue in order to increase the arsenal of new therapeutic agents that can prevent or minimize the neuronal and/or axonal degeneration that occurs. Because of the innumerable, indefinite causes of this disease, there is a tendency to consider it neurological in origin, since the main symptoms lie in this seat. It would be well, however, to start to change the classic point of view.

## Microbiota–Brain Communication

Recent scientific literature has highlighted the close correlation existing between the intestinal microbiota and brain development as well as a correspondence between alteration of the intestinal microbiota and the onset of some neurological pathologies such as anxiety and depression (Strandwitz, 2018), PD, AD (Sun and Shen, 2018), multiple sclerosis (Berer et al., 2017), cerebral ischemia (Nam, 2019), and ASD (Fattorusso et al., 2019). Based on these scientific findings, it is clear that any form of intestinal dysbiosis is able to favor the development of neurological diseases. For just this reason, it is fundamental to know and understand the instruments of dialogue that exist between the intestine and the brain. The intestine can interact with the brain through direct communication, which includes three main mechanisms:

• the enteric nervous system;
• the enteroendocrine cells (EECs) of the gut;
• neurotransmitters produced by the gut microbiota.

### Enteric Nervous System

Functional aspects of the gastrointestinal tract such as peristaltic movements, the transport of substances, and the local flow of blood are all regulated by a network of neuronal ganglia known as the enteric nervous system (ENS) (Furness, 2000; Furness et al., 2004). It is known that the neurons of the ENS communicate with each other using the same “language” as in the CNS (Giuffrè et al., 2020). The ENS consists of two ganglion plexuses composed of neurons and glia that regulate a variety of gastrointestinal functions and are essential for life (Furness, 2006). These plexuses are located between the layers of the gastrointestinal tract and are characterized by about 20 subtypes of neurons that differ by the expression of several neuropeptides (Furness, 2000; Furness et al., 2004). The ENS shares many features with the brain, including the production of neurotransmitters that are used for synaptic transmission, the ultrastructural features present in neuron–glia interaction, and transcriptional programs (Rao and Gershon, 2016; De Vadder et al., 2018). The ENS is capable of operating independently of the brain and spinal cord, but, in healthy subjects, it works in collaboration with them together with input from the vagal, sympathetic, and parasympathetic systems. This is in order to regulate many gastrointestinal functions, such as motility. This direct cross-talk makes the ENS an important target for the pathogenesis of many neurological disorders (Liddle, 2018), and its dysfunction is related to gastrointestinal disorders including severe constipation, anorexia, and gastroparesis. It is also interesting to note that these symptoms are all common in patients with neurological conditions (Chalazonitis and Rao, 2018). The hypothalamic–pituitary–adrenal axis interacts with intestinal epithelium cells via the vagus nerve. Some preclinical studies have shown that the vagus nerve plays a central role in neural communication between the microbes of the intestine and centrally mediated behavioral effects. In particular, following a vagotomy performed early in childhood, these subjects had a lower risk of developing neurological disorders (Svensson et al., 2015). Vagus nerve stimulation is a medical treatment used to treat epilepsy and other neurological conditions and consists in the application of appropriate electrical impulses to the nerve. It is assumed that these electrical impulses exert antiepileptic (Fornai et al., 2011), antidepressant (Sackeim et al., 2007), and anti-inflammatory action by altering the nerve excitability in the cells involved (Breit et al., 2018). A close correlation between the ENS and the microbiota has been demonstrated by the reduced number of enteric neurons and intestinal motility observed in GF mice (McVey Neufeld et al., 2012). In addition important experiments have shown an intrinsically attenuated excitability in afferent primary neurons together with a defective intestinal mucosa in GF mice, despite the development and continuous influx of the ENS (Kabouridis et al., 2015). It is interesting to note that with the administration of the conventional microbiota, the recovery of GF mice saw the density and physiology of the ENS in the intestine normalized (Kashyap et al., 2013). Every microorganism can have a different effect on the ENS: some commensal bacteria may have a local effect interacting with the ENS, while pathogenic bacteria benefit from the ENS by creating an environment more suited to their growth and advantageous for their effects (Giuffrè et al., 2020). The control exercised by the gut microbiota takes place through the vagus nerve and the ENS (Borre et al., 2014; Kaelberer et al., 2018): classic examples are provided by the bacteria *Lactobacillus rhamnosus*, which can modulate anxious behavior, and *Bifidobacterium longum* NCC3001, which exerts anxiolytic effects. It has been shown in mice that these effects are lost after vagotomy (Bercik et al., 2011; Bravo et al., 2011). The microbiota supports the ENS formed at birth and participates in its homeostasis throughout adult life. In fact, in GF mice, it has been shown that the ENS is highly compromised especially in those areas where bacteria are normally found. Increasing evidence shows that some neurodegenerative diseases such as PD can originate in the intestine and spread to the brain by means of the vagus nerve (Klingelhoefer and Reichmann, 2017). The possibility of a close correlation between the dysfunction of the ENS, the microbiota, and the diseases of the CNS has been considered, even if this hypothesis must be further analyzed and deepened.

### Enteroendocrine Cells

EECs reside within the mucosa of the gastrointestinal tract and are electrically excitable. These cells produce more than 20 peptides/hormones in response to signals generated by nutrients, non-nutrient chemicals, food-born toxins, and microorganisms in the bowel lumen (Furness et al., 2013). They influence a variety of physiological functions including digestion and absorption of nutrients, defense responses against harmful/toxic substances, and food aversions (Latorre et al., 2016). These secreted products can act locally, through a paracrine mechanism that activates other EECs, they can be released into the bloodstream, reaching distant targets, or they can act directly on nerve endings near the release site. It is well known that EECs possess many characteristics similar to those of neurons: among these, it is appropriate to remember the receptors of neurotrophins, a family of proteins that induces the survival, development, and function of neurons, and pre- and post-synaptic proteins (Janssen and Depoortere, 2013). The expression of synaptic proteins increases the possibility that the EECs will come into contact with the nerves; there is also a neural circuit that connects the intestinal lumen with the nervous system (Kaelberer et al., 2018). Therefore, it can be said that the EECs represent the first level of integration from the intestinal lumen to the brain capable of generating appropriate functional responses. In particular, the vagal afferent pathways transmit stimuli generated by the EECs to the brain, representing an intermediate station in the bidirectional communication of the brain–intestine axis (Al Omran and Aziz, 2014). A detailed list of EECs, their location, and secreted hormones is shown in Table 1.

**TABLE 1:** EEC subtypes, localization, and secreted hormones (taken and modified from Grochowska et al., 2019).

**Major cell types**	**Localization**	**Major secretory hormones**	**Function**
A (X-like)	Pancreas	Ghrelin	• Appetite control
G cell	Stomach, duodenum	Gastrin	• Gastrin secretion
D cell	Pancreas, stomach, intestine	Somatostatin	• Gastrointestinal hormone release; • Gastrointestinal motility; • Mucosal immunity
L cell	Ileum, colon, duodenum	Glucagon-like Peptide-1; Glucagon-like Peptide-2; Peptide YY (PYY);	• Appetite control; • Gastrointestinal motility; • Energy homeostasis
K cell	Stomach	Gastric inhibitory peptide (GIP)	• Insulin secretion
I cell	Duodenum	Cholecystokinin (CCK);	• Appetite control; • Gastrointestinal motility; • Bile acid and digestive enzyme release; • Mucosal immunity
Enterochromaffin cell	Small intestine, colon, appendix	Serotonin (5-HT)	• Appetite control; • Gastrointestinal motor and secretory function; • mucosal immunity
N cell	Small intestine	Neurotensin	• Gastrointestinal motility; • Mucosal immunity
M cell	Peyer’s patches	Motilin	• Gastrointestinal motility
S cell	Small intestine, duodenum	Secretin	• Acidity; • Body fluid homeostasis
Enterochromaffin-like cell	Gastric glands	Histamine	• Acidity; • Mucosal immunity

### Neurotransmitters Produced by the Gut Microbiota

The intestinal microbiota is also able to synthesize many neurotransmitters such as dopamine, serotonin, norepinephrine, and δ-amino butyric acids (GABA) that also exercise their own effects on the brain. For example, *Bifidobacterium infantis* has been shown to elevate tryptophan levels in blood plasma and thus influence central serotonin transmission; *Lactobacillus* and *Bifidobacterium* can produce GABA; *Escherichia*, *Bacillus*, and *Saccharomyces* spp. can produce noradrenaline; *Candida, Streptococcus, Escherichia*, and *Enterococcus* spp. can produce serotonin; *Bacillus* can produce dopamine; *Lactobacillus* can produce acetylcholine (Lyte, 2014). These neurotransmitters can go through the mucous layer of the intestine and enter the bloodstream, but they are not able to cross the blood–brain barrier. The impact on brain function, therefore, could be indirect by acting on the enteric nervous system (Dinan and Cryan, 2017). SCFAs, which include butyrate, propionate, and acetate, are essential metabolic products of gut microbial activity and may affect the brain, energy balance, and metabolism (Dinan et al., 2015). In addition, SCFAs have neuroactive properties. High doses of propionate, in young rats, induced a neuroinflammatory response and behavioral alterations while butyrate reduced the depressive behavior, exerting an effect on the CNS (Foley et al., 2014). To date, it is known that SCFAs act preferably as epigenetic modulators through histone deacetylases (Stilling et al., 2014). The gut–brain axis has another signaling pathway that involves immunity through cytokines. Cytokines, produced in the intestine, can flow into the bloodstream and, under altered conditions, can affect areas of the brain such as the hypothalamus (El Aidy et al., 2014).

## Discussion and Conclusion

This review summarizes the knowledge, to date, on the importance of the intestinal microbiota and how the intestinal bacterial component manages to communicate with the brain (Quigley, 2017). In particular, the continuous cross-talk existing between the intestine and the brain and how the intestinal microbiota maintains constant and continuous interaction with the nervous system is highlighted. Intestinal dysbiosis, in fact, is directly involved in many brain disorders (Westfall et al., 2017; Russo et al., 2018). The causes of neurodegenerative diseases are still unknown, but it is certain that several factors including genetics, lifestyle, and aging play key roles. For example, healthy intestinal barrier function seems crucial for maintaining neurological health (Di Meo et al., 2018) and studies have been conducted to assess microbial composition in patients suffering from neurodegenerative diseases (Mohajeri et al., 2018). The microbiota is able to determine the severity of neurodegenerative diseases through two mechanisms:

• immuno-mediated neurodegeneration (Chen et al., 2016; Dombrowski et al., 2017);
• direct effect of metabolites (GABA, histamine, dopamine, norepinephrine and serotonin) on cells of the CNS (Corasaniti et al., 2007; Bano and Ankarcrona, 2018; Strandwitz, 2018).

In PD, it is interesting to observe that the compromised parts are the most caudal of both the CNS and the enteric nervous system (Clairembault et al., 2015). For this reason, the intestine and its effects on the CNS were investigated and many researchers are trying to evaluate whether PD begins in this organ; what is certainly undisputed is the role of the gut microbiota in the pathology of PD. In humans there are data showing that in the pathophysiology of PD, truncal vagotomy reduces the risk of PD (Perez-Pardo et al., 2017; Lionnet et al., 2018). Several alterations in the composition of the microbiota have been found in patients with PD which include a reduction in Firmicutes, *Clostridium saccharolyticum, Clostridium leptum*, and *Faecalibacterium.* In addition, a reduction of *Prevotella* occurs in the early stages of PD and could work as a biomarker for PD (Keshavarzian et al., 2015; Hill-Burns et al., 2017; Hopfner et al., 2017; Petrov et al., 2017). In the light of this scientific evidence, we can state that the bacterial composition of the colon may be predictive for PD (Li et al., 2017), although further assessments should be conducted.

Several studies in recent years have been carried out and have highlighted the involvement of the intestinal microbiota in the onset and pathophysiology of AD (Hu et al., 2016; Jiang et al., 2017). In particular, significantly decreased *Clostridium leptum* and *Clostridium saccharolyticum* were observed in AD as well as an increased Bacteroidetes phylum and *Alistipes* genus (Gerhardt and Mohajeri, 2018). A disbiotic intestinal microbiota produces and releases a mixture of metabolic products that increase the production of cytokines and inflammatory mediators. These compounds induce the amyloid aggregation present in AD by accumulating Aβ, hyperphosphorylating the Tau protein, and inducing chronic inflammation in the brain. In addition, during aging, regenerative capacities are reduced, leading to an increase in neurodegenerative processes and the clinical manifestations of dementia (Penke et al., 2017).

Among neurological disorders, three pathological conditions have been examined that occur very commonly in the population, which are closely related to the alteration of the microbiota and which can appear at early or very early times in life. These three pathologies are anxiety and depression, autism spectrum disorders, and multiple sclerosis, and despite having completely different specific characteristics, they have the following points in common:

• are diseases related to the malfunction of the nervous system;

• are closely related to intestinal dysbiosis;
• occur in pathological form in a very early period of life: the phenomena of anxiety and depression can occur as early as childhood or adolescence, but also at any time in an individual’s life. Autism spectrum disorders develop and appear during the first year of life. Multiple sclerosis appears particularly in young women (female:male ratio = 3:1), especially in those women who have had intestinal disorders since birth (Sauma and Casaccia, 2020).

This review does not intend to focus attention only on the close gut–brain communication, which is already well-known and studied in-depth, but intends to look at this problem from an identical but opposite perspective, and that is: “What if these pathologies actually had a non-neural onset?” and “Is it possible that these pathologies develop due to an altered microbial composition in the gut?”

If so, an evaluation of the intestinal microbial composition would be fundamental as an early preventive tool against brain diseases. The moment of intestinal colonization, during the very early stages of life, could be fundamental and determinative; in fact, if this process were to occur inadequately, an imbalance in the composition of the microbiota would be set off, which could persist throughout life. So, another vital question is: “How important is the breastfeeding process?” This type of feeding could provide the infant with an already mature and balanced “immune culture” capable of reacting promptly to a wide variety of external pitfalls.

In this direction, it could try to cure not the neurological cause, but directly correct the composition of the microbiota. So, whenever there are any dysbiosis conditions present, it would be desirable to:

• carry out prenatal and neonatal screening to find out the exact composition of the microbiota and in case of alterations correct it by using specific prebiotics and probiotics;
• repeat this screening periodically in order to identify the onset of intestinal dysbiosis;
• start to consider these pathologies as intestinal diseases rather than nervous diseases;
• consider the intake of substances of natural origin capable of establishing a correct oxidative status of the organism.

**FIGURE 1:**
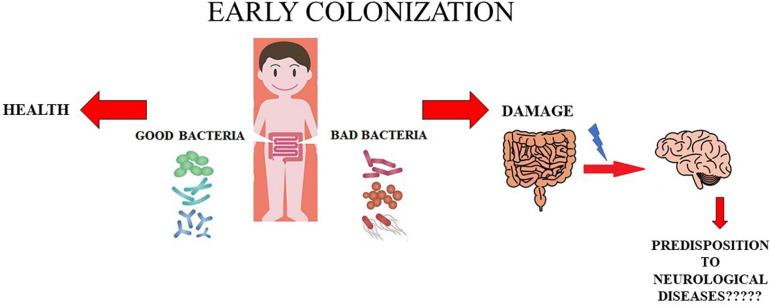
Role of early colonization. In this cartoon the role of early colonization is shown. In particular, when the colonization is caratecrized by appropriate and beneficial bacteria the health status is mantained. On the contrary, whether bad bacteria early colonize the intestinal tract we can have damages in this organ and probably probably a predisposition to onset of some neurological diseases.

Further studies and insight into this topic are needed to change the point of view from which these issues are being observed and studied. A conclusive and definitive evaluation is indispensable before automatically assuming that anxiety and depression, ASD, and MS have a strictly neural origin; the hypothesis that intestinal dysbiosis could be the real culprit should be investigated thoroughly. This hypothesis is represented in Figure 1.

## Author Contributions

JM and VMo conceptualized and designed the manuscript. JM, MG, and VMo wrote the manuscript. RM, FO, SN, FB, VMu, CC, FS, MS, EP, SR, MZ, and CM revised the manuscript critically. All authors contributed to the article and approved the submitted version.

## Conflict of Interest

The authors declare that the research was conducted in the absence of any commercial or financial relationships that could be construed as a potential conflict of interest.
