# Supplementary figures and images for: The Contribution of Gut Microbiota–Brain Axis in the Development of Brain Disorders
Source: Front Neurosci. 2021 Mar 23;15:616883. doi: 10.3389/fnins.2021.616883 (PMC8021727; doi:10.3389/fnins.2021.616883)

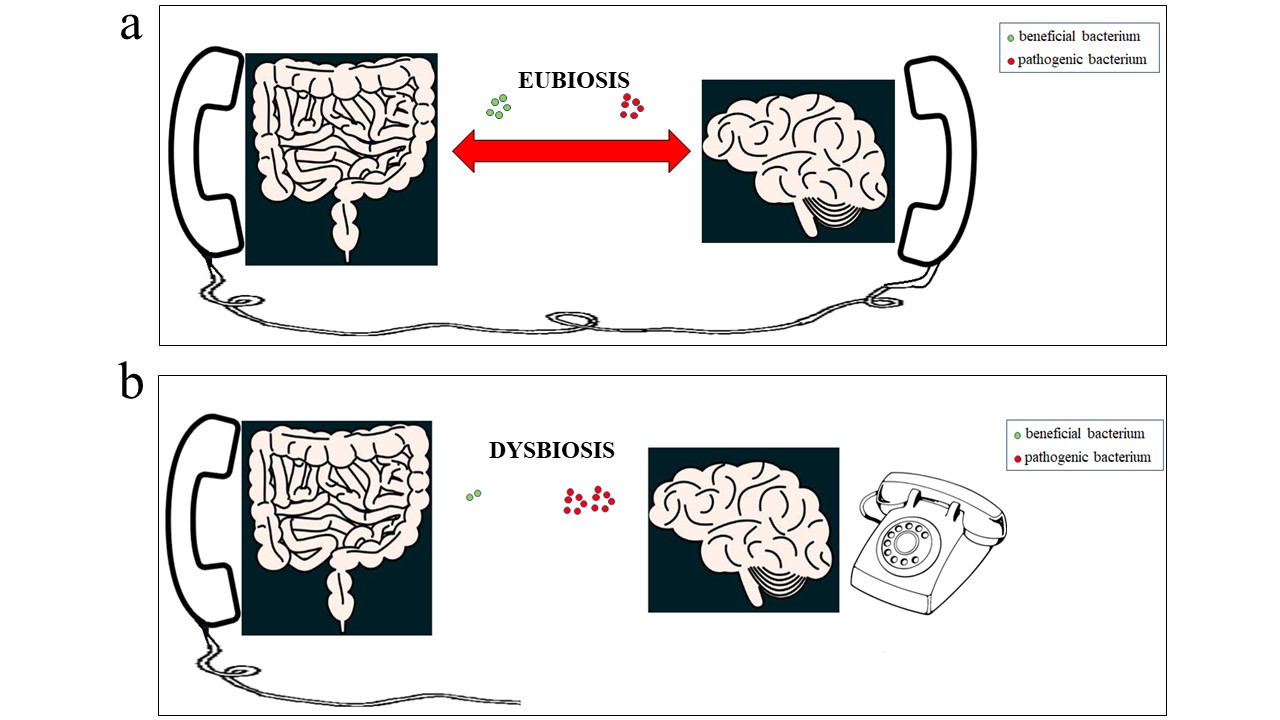

Supplement: Supplementary file 1 [file Image_1.JPEG]
